# Hospital protocol for universal use of coiling to all brain aneurysms in non-university setting: Is it too late to be corrected?

**DOI:** 10.4103/2152-7806.63914

**Published:** 2010-05-31

**Authors:** Ramsis Ghaly

**Affiliations:** Ghaly Neurosurgical Associates, Aurora, IL 60504, USA

Sir,

Not long ago in the 1990s during my neurosurgery training at University of Illinois at Chicago, interventional techniques for brain aneurysms were introduced. I was fortunate to learn under Dr. James Ausman, father of vascular neurosurgery, and Dr. Gerard Debrun, father of interventional neuroradiology. We learned that every case should be presented to both disciplines, and to then do whatever is considered in the best interest of the patient, whether surgical clipping or coiling, or both. The consideration was never “coil all brain aneurysms,” nor to reduce the bar in order to avoid surgery. Discussions were held daily about aneurysm cases; the discussions were professional, enjoyable, and respectful between the surgeon and interventionalist.

A 26-year-old woman presented with a history of left unruptured carotid terminus aneurysm (7 mm) with wide neck. The patient was referred for cerebral angiography to a non-teaching, but large regional medical center. After the completion of the cerebral angiography, an interventional neuroradiologist placed the patient on Plavix and automatically scheduled the patient for coiling and possible “stenting” of the carotid terminus aneurysm. The patient called the neurosurgeon and assumed that “coiling” was what the neurosurgeon recommended as “standard practice.” In contrast, it was neither what the neurosurgeon recommended, nor was it what the patient desired. The patient was then educated and began to understand both techniques. Then, she expressed her decision to pursue “surgical clipping.” The patient was subsequently interrogated by the interventionalist, and the neurosurgeon was questioned in regard to the “deviation” of hospital “protocol and policy.” A second opinion was obtained from a well-known vascular neurosurgeon, who also agreed that “surgical clipping” was the best option for this patient. The patient underwent a surgical clipping, was cured from the aneurysm, and recovered from the surgery with no neurological deficits. She is currently working and has no further worries.

Currently, it is a common practice in community hospitals that all brain aneurysms, both ruptured and unruptured, be handled by coiling and to have the neurosurgeon “on call” when complications arise. This practice is generally written as a “written protocol” or a policy. Deviation from this practice will place the neurosurgeon in hardship and will be evaluated as an action against the “hospital policy.” In fact, the interventional neuroradiologist feels that no options should be given the patient regarding coiling or surgical clipping. The patient then finds himself/herself subjected to treatment that is considered “standard practice.” It appears that there are no options available to the patients anymore. The success of the interventionalist is based on how many of these aneurysms can be coiled, not necessarily what is best for the patient. Sadly, the accepted practice is not to raise the bar to “cure” the aneurysm, but to “control” the aneurysm. Many times neurosurgeons, especially those employed by the hospital, are finding themselves unable to voice what is right for the patient, but what is right for the “protocol.” Obviously, this practice is not “standard of care” and is not what vascular neurosurgeons and followers utilizing “evidence-based practice” will do. Unfortunately, it is common in community hospitals and not in teaching hospitals, where experienced vascular neurosurgeons are present and can voice their concerns. It also appears that the community hospitals have found a selling “niche” to coil brain aneurysms and avoid brain surgery, and ignore what is best for the patient in the long run. It also calls into question the quality control measures for the brain aneurysm in community hospital care. Are we serving the patient by misleading the facts, using their naivete, and presenting the attraction of “avoiding” brain surgery? Furthermore, the practice of “coiling all aneurysms” has limited many referrals for aneurysms to regional centers. Subsequently, many neurosurgery programs are seeing decreases in aneurysm surgery. Neurosurgery residents are going out of state and even sometimes overseas to learn about aneurysm surgery. I believe it is the right time for our neurosurgeons to strongly oppose this growing practice of a “universal protocol of coiling all brain aneurysms.”

